# A C-terminally truncated mouse Best3 splice variant targets and alters the ion balance in lysosome-endosome hybrids and the endoplasmic reticulum

**DOI:** 10.1038/srep27332

**Published:** 2016-06-06

**Authors:** Lichang Wu, Yu Sun, Liqiao Ma, Jun Zhu, Baoxia Zhang, Qingjie Pan, Yuyin Li, Huanqi Liu, Aipo Diao, Yinchuan Li

**Affiliations:** 1 Department of Animal Sciences and Technology, Qingdao Agricultural University, Qingdao, China; 2 College of Biotechnology, Tianjin University of Science and Technology, Tianjin, China

## Abstract

The Bestrophin family has been characterized as Cl^−^ channels in mammals and Na^+^ channels in bacteria, but their exact physiological roles remian unknown. In this study, a natural C-terminally truncated variant of mouse Bestrophin 3 (Best3V2) expression in myoblasts and muscles is demonstrated. Unlike full-length Best3, Best3V2 targets the two important intracellular Ca stores: the lysosome and the ER. Heterologous overexpression leads to lysosome swelling and renders it less acidic. Best3V2 overexpression also results in compromised Ca^2+^ release from the ER. Knocking down endogenous Best3 expression in myoblasts makes these cells more excitable in response to Ca^2+^ mobilizing reagents, such as caffeine. We propose that Best3V2 in myoblasts may work as a tuner to control Ca^2+^ release from intracellular Ca^2+^ stores.

The Bestrophin family was initially regarded as a candidate Ca^2+^ activated Cl^−^ channel (CACC)^1^,^2^,^3^,^4^. In 2008, people showed that the Anoctamin was the typical CACC^5^,^6^. Thus, the *in vitro* roles of Bestrophins became more confusing at that time. Bestrophin was also revealed to be a putative candidate volume-regulated ion channel^7^,^8^, which conducts HCO3^−^ or gamma amino butyric acid (GABA)^9^,^10^,^11^. Recently, the crystal structure of Bestrophin was generated and revealed a clear channel core for Cl^−^ in human Best1^12^. The crystal structure of human Best1 also displayed a Ca^2+^ clasp or Ca^2+^ bowl in aa 300–304^12^. However, the successful generation of crystals of the bacterial Bestrophin homolog revealed that it is a Na^+^ channel^13^. The Bestrophin family may have diverse or versatile roles.

The Bestrophin family has 4 members in humans and 3 or 4 Bestrophin paralogs in other animals^14^. *BEST1* or *VMD2* (vitelliform macular dystrophy 2) has been linked to an autosomal dominant form of juvenile blindness known as Best vitelliform macular dystrophy^15^,^16^, which was regarded as a type of lysosome storage disease. Unfortunately, people still do not understand the direct correlation between Best1 and the lysosomes^14^. However, a role for Best1 in maintaining intracellular Ca^2+^ balance has been proposed in RPE cells^17^. Best2 was shown to be involved in generating aqueous flow and controlling the intraocular pressure in eyes^18^,^19^. Best3 is very abundantly expressed in muscle tissues, along with diverse splice variants^20^, but their exact roles in muscles remain largely undefined.

In this report, a natural splice variant (Best3V2) of mouse Best3 without the long C-terminus (an alternative splice variant after amino acid L360 that introduces a premature stop codon at amino acid 364 due to a frame shift) was cloned from mouse myoblasts. This splice variant was capable of inducing cell vacuolization in cultured cell lines. Therefore, these vacuoles can help track their recruitment to certain intracellular organelles and facilitate the monitoring of the factors contributing to vacuolization. More importantly, this splice variant provides new insights into its role in myoblasts.

## Results

### Muscles express novel Best3 splice variants and one splice variant induces vacuoles in cell cytosol

**Figure 1:**
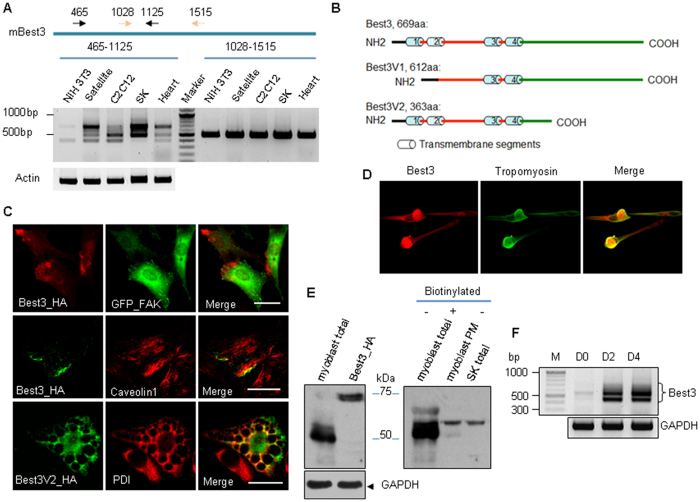
The expression of Best3 in myoblasts, muscle tissues and fibroblasts. (**A**) The expression of mouse Best3 in NIH3T3, myoblasts differentiated from satellite cells, myoblasts differentiated from C2C12 (4 days after confluency), skeletal muscle (SK) and heart was assessed by RT-PCR. Two sets of primers for mouse Best3 sequence (NM_001007583) were used. Alpha actin was used as an internal control. (**B**) Schematic presentation of full-length mouse Best3 and 2 alternatively spliced variants V1 and V2 (Best3V2). (**C**) Distinct distribution patterns of full-length Best3 and Best3V2. The ER was stained with PDI, the membrane ruffles were stained with an anti-caveolin 1 antibody, and some focal adhesions were visualized with GFP_FAK. Scale bar, 20 μm. (**D**) The expression of endogenous Best3 in differentiating myoblasts derived from satellite cells. The cells were also co-stained with a tropomyosin antibody to determine the levels of endogenous Tropomyosin. (**E**) The size of the endogenous Best3 proteins and its targeting to the plasma membrane in differentiating C2C12 myoblasts. Plasma membrane targeting was monitored via cell surface protein biotinylation. Whole proteins of skeletal muscle (SK) and C2C12 myoblasts were used as controls. (**F**) Best3 expression was analyzed in myoblasts differentiating from C2C12 cells on day 0 (D0), day 2 (D2) and day 4 (D4) by RT-PCR. M, DNA marker. The experiments were repeated 3 times.

Best3 was extensively expressed in skeletal muscles, cardiac muscles, myoblasts and NIH3T3 fibroblasts (Fig. 1A). Two unique splice variants of Best3 were cloned from differentiating C2C12 myoblasts and mouse skeletal muscles (Fig. 1B). Full-length Best3 was predominantly located in plasma membrane folds with a polarized distribution pattern and was partially colocalized with caveolin 1 (Fig. 1C). The C-terminally truncated Best3V2 splice variant was predominantly located in intracellular organelles and partially colocalized with the ER protein PDI in NIH3T3 cells. However, this splice variant is expressed at very low levels in the tested cells. Best3V2 also induced vacuolization in NIH3T3 cells and much stronger vacuolization in HeLa cells (Fig. S1). In this report, we mainly focus on Best3V2, due to its ability to induce vacuoles, which makes it easier to visualize.

The Best3V2 vacuoles were distinct from the vacuoles induced by Clc3s (a N-terminally truncated splice variant of Clcn3) or VPS4(E228Q) (mutation of Vacuolar Protein Sorting 4 Homolog A, VPS4a) in size, morphology, lifetime, fusion properties and adverse effects on cells (Fig. S1), suggesting that these 3 vacuoles had distinct origins. The vacuoles induced by Best3V2, Clc3s and VPS4(E228Q) are named Vac-V2, Vac-Clc3s and Vac-VPS4(E228Q), respectively. The Best3 protein was also endogenously expressed in myoblasts that were differentiating from satellite cells (Fig. 1D). The Western blots of the biotinylated plasma membrane proteins showed that the Best3 proteins were expressed at high levels in differentiating myoblasts (Fig. 1E). However, most of the proteins were smaller than the full-length Best3 protein, and only one band (approximately 52 kDa) was targeted to the plasma membrane. The Best3 splice variants were expressed at very high levels at the start of C2C12 cell differentiation. However, its role in myoblasts is still unknown.

### The Best3V2-induced vacuoles are swelling lysosomes

**Figure 2:**
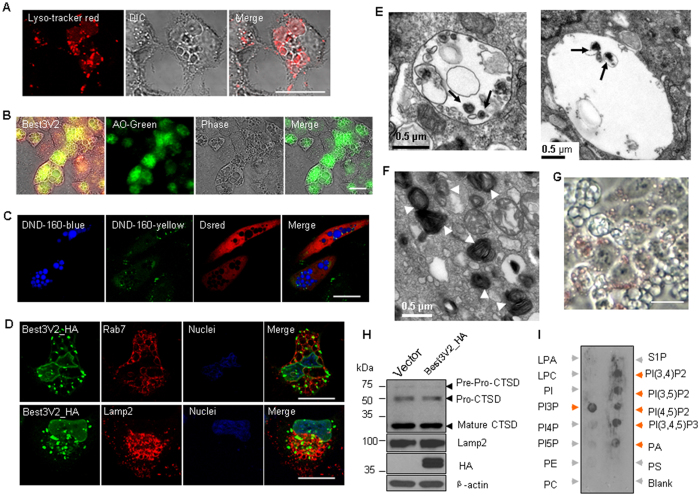
Vac-V2s are swelling lysosomes. (**A**) Live cell imaging of the lysosome dye LysoTracker Red within the Vac-V2s. The DIC images showed the outlines of the Vac-V2s. (**B**) Images of the AO (5 μg/ml)-stained Vac-V2s in live cells. (**C**) The Vac-V2s were stained with Lysosensor Yellow/Blue DND-160 (5 μM) and showed emission wavelength shift from yellow (shown in green) (low pH) to blue (higher pH). Cotransfected DsredN1 indicated the outline of the positively stained cells. The green puncta denote the normal lysosomes. (**D**) Partial co-localization of Best3V2_HA with endogenous Rab7 or Lamp2. (**E**) The transmission electron microscopy (TEM) images showed the morphology of the Vac-V2s in HeLa cells (36 h). Electron dense granules were occasionally observed within the vacuole lumen (arrows). The left image showed a small, emerging Vac-V2 with a clear single membrane. The right image showed a large Vac-V2. (**F**) The TEM images showed the multilamellar bodies (MLB) (white arrow) in NIH3T3 cells that were induced by Best3V2. (**G**) Oil red O staining showed the undigested lipids (red) within the Vac-V2 lumen (48 h) in HeLa cells. (**H**) The effect of Best3V2 on the maturation of the lysosome enzyme cathepsin D (CTSD). (**I**) PIP strips were blotted with His_HA-tagged aa 114–153. The positively stained phospholipid dots were shown by the orange arrows and negative dots were shown by the grey arrows. Scale bar, 20 μm. The experiments were repeated 3 times.

Vacuoles may not be the physiological consequence of endogenous Best3V2 expression, but the vacuoles provide clues that can be used to track its subcellular targeting. Live cell imaging showed that Vac-V2s were lysosomes, because the vacuoles of the HeLa cells were filled with small LysoTracker Red granules (Fig. 2A). To characterize the pH within vacuoles, we stained these cells with acridine orange (AO) or the biphasic lysosome dye Lysosensor DND160. AO labeling showed that the lumens of the Vac-V2s were acidic and also exhibited a strong green shift, a sign of a less acidic lumen (Fig. 2B). Staining with the biphasic pH-sensitive dye Lysosensor DND 160 also confirmed that the Vac-V2s were less acidic than the lysosomes (Fig. 2C). We then labeled the vacuoles with endogenous Rab7a or Lamp2 to discriminate the origin of the Vac-V2s. The fluorescence images showed that the membranes of most Vac-V2s in HeLa cells were strongly stained by Rab7a and, to lesser extent, by Lamp2 (Fig. 2D). Noticeably, Best3V2_HA was located in the nuclear envelope and in the vacuole membrane, in contrast to full-length Best3, which was mainly located in the plasma membrane folds shown in the preceding image. A considerable number of Lamp2-positive granules were negative for Best3V2s, suggesting that primary lysosomes were not the origin of the Vac-V2s. Rab7-positive vesicles are usually late endosomes or lysosomes (including endosome-lysosome hybrids and autolysosomes)^21^,^22^,^23^. The TEM images showed that the Vac-V2s contained electron dense lysosome granules with undigested membranous structures, further confirming the compromised digestion in the less acidic lumen of the Vac-V2s in HeLa cells (Fig. 2E). Noticeably, the TEM images also showed that the Best3V2-transfected NIH3T3 cells were filled with a large number of multilamellar lysosomes instead of vacuoles, which may explain why the NIH3T3 cells contained fewer Vac-V2s than the HeLa cells (Fig. 2F, Fig. 1S). Therefore, the cells that underwent vacuolization or formed multilamellar lysosomes upon Best3V2 overexpression varied depending on the cell types. However, the common finding for both cell types was compromised lysosome digestion, and lysosomes were the target of BestV2 in both cell types. Vacuoles or multilamellar bodies may not be the physiological consequence of endogenous Best3V2 expression but may result from overexpression. Oil red O staining further confirmed that a number of incompletely digested lipids accumulated within the Vac-V2s in HeLa cells (Fig. 2G). However, the reduced acidification in lysosomes induced by Best3V2 did not affect the normal maturation process of the lysosomal enzyme cathepsin D (CTSD) in HeLa cells (Fig. 2H), suggesting that the enzyme trafficking and maturation through the Golgi apparatus, endosome and ultimately to the primary lysosome was not impaired by Best3V2. In this context, the primary lysosomes were less likely to undergo vacuolization, and the endo-lysosome hybrid may be one candidate target. More interestingly, the phospholipid-binding domain on the cytosolic loop of Best3V2 was able to bind PI_3_P and PI_(3,5)_P_2_ (Fig. 2I), which are two specific phospholipids of the endo-lysosome system^24^. Recombinant aa 114–153 of Best3V2, which are located within the cytosolic loop between TM2 and TM3, were able to bind to phospholipids, such as PI_3_P, PI_(3,4)_P_2_, PI_(3,5)_P_2_, PI_(3,4,5)_P_3_ and PA. This result further implies that Best3V2 has the ability to bind endosome-lysosome vesicles. A double mutation at aa 114–153 (R125Q and R126Q) abolished phospholipid binding (data not shown).

In summary, regarding the origin of the Vac-V2s, the evidence indicates that they originate from the lysosomes, particularly the endo-lysosome hybrids.

### Failure to reform lysosome-endosome hybrids leads to vacuolization

**Figure 3:**
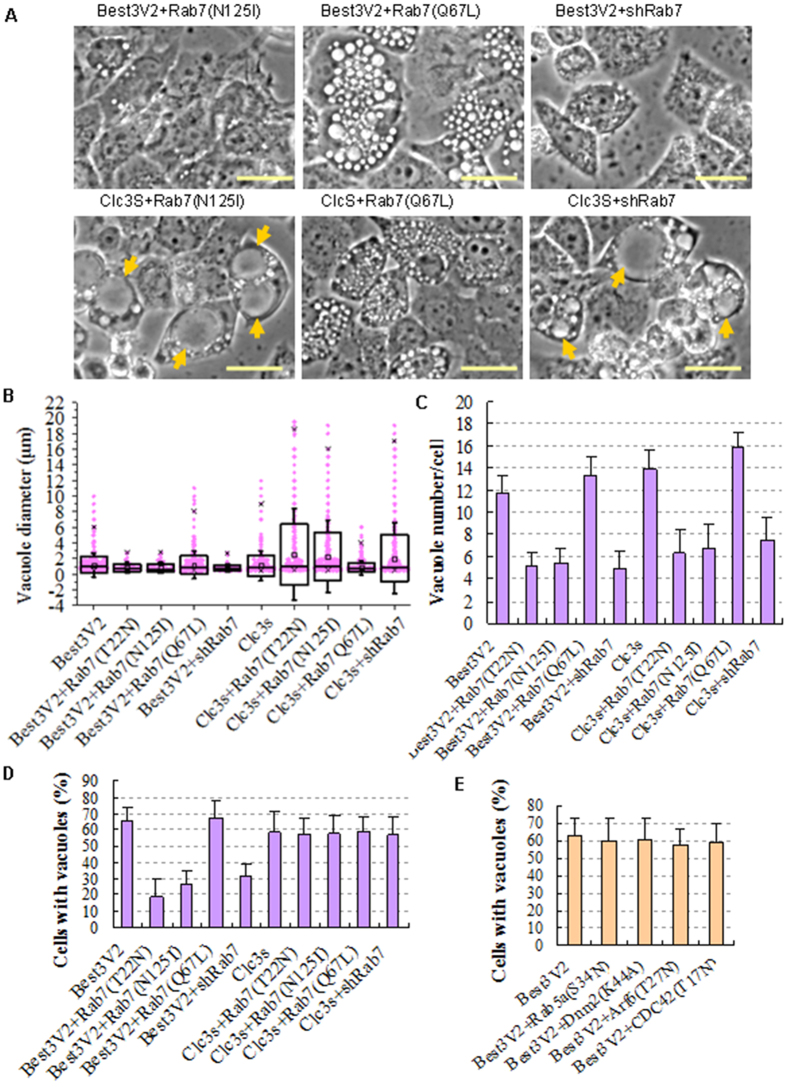
Blocking Rab7 inhibits Vac-V2 formation. (**A**) Representative phase contrast images showing the opposite effects of Rab7 on Vac-V2 and Vac-Clc3 formation in HeLa cells. The arrows showed extremely large Vac-Clc3s that were induced by inhibiting Rab7. Scale bar, 20 μm. (**B**–**D**) The impacts of dominant negative Rab7 mutants, the constitutively active Rab7 (Q67L) mutant and shRab7 on the vacuole size, number of cells with vacuoles, and average vacuole number per cell, which were induced by Best3V2 or Clc3s as in (**A**). (**B**) Scatter plots showing the distribution of vacuole diameters induced by the indicated plasmids. (**C**) Scatter plots showing the average vacuole number per cell. (**D**) Scatter plots showing the percent of cells with vacuoles. One hundred cells were randomly selected for counting and calculation. (**E**) Vac-V2 formation in response to the inhibition of early endocytosis by the 4 dominant negative mutants, respectively. The error bars denote the S.D. values. The experiments were repeated 3 times.

We next monitored whether these vacuoles were generated from late endosome-lysosome hybrids. The fusion process of endosome and lysosome should be blocked or enhanced to block or increase the formation of endosome-lysosome hybrids, respectively. Rab7 is the key protein that mediates this fusion process^24^. Therefore, dominant negative mutants (Rab7a(T22N), Rab7a(N125I)) and a constitutively active mutant (Rab7a(Q67L)) of Rab7a and shRNA constructs (shRab7a) were applied to either block or enhance Rab7a-mediated fusion between endosomes and lysosomes. Clc3s was also used as a parallel control to show the effect of blocking or enhancing late endosome-lysosome fusion. Unfortunately, our data showed that blocking or enhancing Rab7a activity had contrasting effects on the formation of Vac-V2s and Vac-Clc3s (Fig. 3A). Rab7a inhibition decreased the size of the formed Vac-V2s (Fig. 3B–D). In contrast, Rab7a inhibition significantly increased the size of the Vac-Clc3s and Rab7a(Q67L)-enhanced Rab7a-mediated fusion abolished the generation of large diameter Vac-Clc3s, but not the small-sized Vac-Clc3s. The results suggest that the large sized Vac-Clc3s are exclusively derived from fusion among endosomes, and upon fusion with lysosomes, the Vac-Clc3s are degraded by the lysosomes. These data clearly indicate that the Vac-V2s are mainly derived from endosome-lysosome hybrids, whereas the Vac-Clc3s are exclusively generated from endosomes. Enhancing the fusion of late endosomes with lysosomes prevented fusion among Vac-Clc3s and thus eliminated the formation of large Vac-Clc3s. This result is different from a previous report showing that the vacuoles generated by Clc3s were lysosomes^25^. Furthermore, TEM confirmed the fusion of a large Vac-Clc3 with a small, emerging Vac-Clc3 (Fig. S2).

To rule out the direct involvement of early endosomes in inducing Vac-V2s, we selectively blocked Rab5, Dynamin 2 (Dnm2), Arf6 or CDC42 with their corresponding dominant negative constructs: Rab5a(S34N), Dnm2(K44A), Arf6(T27N) or CDC42(T17N), respectively. As expected, these mutations failed to block Vac-V2 formation (Fig. 3E).

Therefore, by comparing the generation of Vac-V2s and Vac-Clc3s by blocking or enhancing endosome and lysosome fusion, we show that the Vac-V2s are derived from endosome-lysosome hybrids and Vac-Clc3s are formed from endosomes.

### ER vacuoles induced by mutating the phospholipid binding domain or by adding an ER retention signal to Best3V2 and their properties compared with vacuoles induced from lysosomes

**Figure 4:**
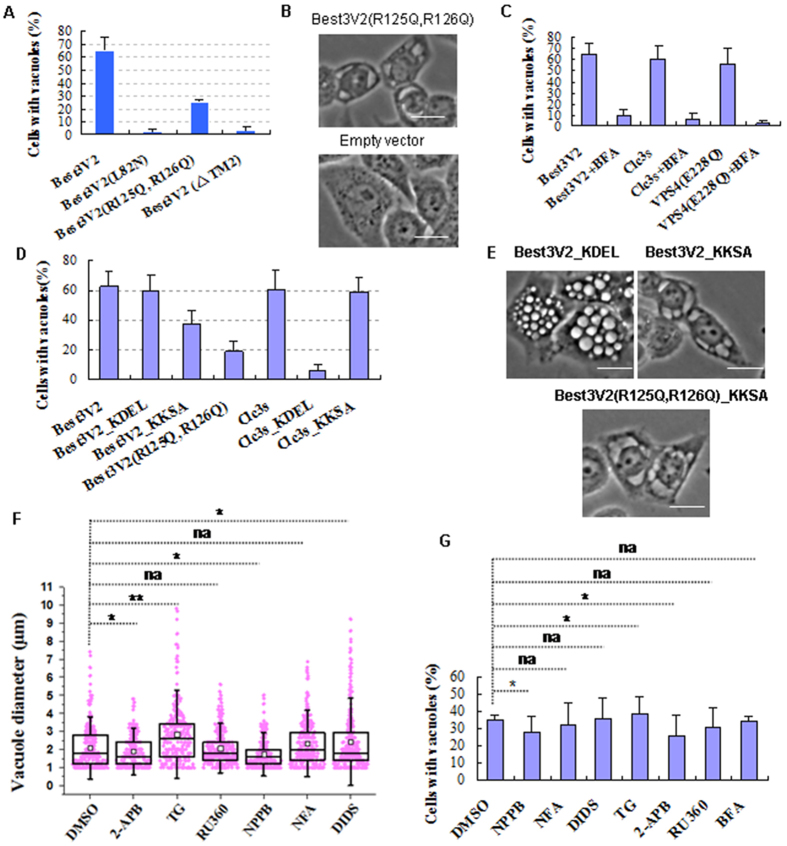
Retention of Best3V2 in the ER also results in ER vacuolization. (**A**) Mutation of TM2 (L82N), mutation of 2 amino acids (R125Q and R126Q) in its phospholipid binding domain, or deletion of TM1 and TM2 (natural splice variant V1 of Best3) abolished Vac-V2 formation in HeLa cells. (**B**) Low-diopter vacuoles induced by Best3V2(R125Q, R126Q) in HeLa cells. Cells that were transfected with the empty vector were used as the control. (**C**) Bafilomycin A1 (BFA) strongly decreased vacuole formation. The same treatment was also applied to the Clc3s and VPS4(E228Q) as parallel controls. (**D**) Distinct impact of the ER luminal side retention signal _KDEL and ER cytosolic side retention signal _KKXX (KKSA) on the generation of Vac-V2 or Vac-Clc3s. (**E**) Morphology of vacuoles induced by Best3V2_KDEL, Best3V2_KKSA or Best3V2(R125Q,R126Q)_KKSA in HeLa cells. (**F**) The impact of ion channel blockers on Vac-V2 formation. One hundred vacuole-positive HeLa cells were used for counting. *p < 0.05. **p < 0.01. (**G**) Several channel blockers inhibited Best3V2_KKSA-induced ER vacuole formation in Hela cells. The error bars denote the S.D. values. Scale bar, 20 μm. The experiments were repeated 3 times.

Then, we determined whether mutation of the phospholipid binding domain, aa 114–153, or the putative channel core of Best3 can abolish the swelling of endosome-lysosome hybrids, because aa 114–153 were shown to bind PI3P and PI(3, 5)P2. Moreover, according to the channel structure of human Best1 or bacterial Bestrophin^12^,^13^, a segment of transmembrane domain 2 (TM2) formed the putative conducting channel core. Interestingly, mutation of transmembrane domain 2 (TM2) (TM2 (L82N)) and Best3V2 (ΔTM2) derived from a natural alternative splice variant, Best3V1, without TM2 abolished the formation of bright round vacuoles in HeLa cells (Fig. 4A). Mutation of the phospholipid binding domain, aa 114–153 (Best3V2(R125Q, R126Q)), abolished the formation of the bright round vacuoles, and, instead, low-diopter vacuoles formed (morphology was shown in Fig. 4B). More importantly, the low-diopter vacuoles were not blocked by the H^+^ pump blocker BFA. However, the bright vacuoles induced by Best3V2 or other vacuoles derived from acidic endosome-lysosome systems, i.e., Vac-Clc3s and Vac-VPS4(E228Q), can be effectively blocked by BFA, suggesting that the two morphologically distinct vacuoles are derived from different organelles in HeLa cells (Fig. 4C).

We then sought to determine which organelles were induced by Best3V2 (R125Q, R126Q) and then generated the low-diopter vacuoles. We ruled out the contribution of the acidic endo-lysosome system, and the ER, a neutral pH organelle, became our focus. We then added an ER retention signal to Best3V2 and tested whether the retention of Best3V2 in ER also induced the formation of low-diopter vacuoles similar to Best3V2(R125Q, R126Q). Two ER retention signals were introduced. One is located in the cell cytosol and has the _KKXX consensus sequence^26^. The other is within the ER lumen and has the well-known _KDEL consensus motif^27^. According to the topology of Best1, the two termini are facing the cell cytosol^28^,^12^,^13^. Therefore, we fused _KKXX (_KKSA) to its C-terminus to obtain Best3V2_KKSA. Our data showed that _KKSA was indeed able to eliminate the formation of the bright round vacuoles, but not _KDEL (Fig. 4D,E). For Best3V2_KKSA, instead of bright round vacuoles, a large number of low-diopter vacuoles were formed in the perinuclear region (Fig. 4E), suggesting that the vacuoles induced by retention in the ER were similar to Best3V2 (R125Q, R126Q). In contrast, Clc3s_KDEL with the luminal retention signal nearly eliminated vacuolization and did not form any low-diopter vacuoles, but Clc3s_KKSA had a similar vacuolization capacity as Clc3s, suggesting that its C-terminus was within ER luminal side, which is opposite to full-length Clc3. According to the topology of Clc3s, both of its termini should be inserted toward the cytosolic side^28^. The result strikingly suggests that Clc3s is inserted in the organelle membrane in a reverse orientation when the N-terminal sequence is naturally deleted, and, alternatively, the C-termini of Clc3s turns to face the ER lumen. Therefore, the natural splice variant Clc3s completely alters the insertion topology in the organelle membrane, which enriches the function of Clc3. This interesting and unique phenomenon for channel genes extends their functions by undergoing alternative splicing to change their insertion topology into membrane. Notably, Clc3s_KDEL retention in the ER did not induce any visible vacuolization, suggesting that not all ion channels display channel activity in ER when they are confined to this organelle. This parallel comparison between the ER retention of Clc3s and Best3V2 further emphasizes that the low-diopter vacuoles formed by Best3V2 (R125Q, R126Q) and Best3V2_KKSA originated from the ER and resulted in an ion imbalance in ER. However, Clc3s retention in the ER is not capable of inducing the formation of any visible vacuoles in the ER. Additionally, our data also suggest that aa 114–153 are critical for Best3V2 targeting and binding to the lysosome.

However, the anion channel blockers NFA, NPPB and DIDS did not substantially inhibit the formation of either the bright or low-diopter vacuoles (Fig. 4F,G). Although only NPPB inhibited both types of vacuoles (p < 0.05), it is not clear whether its inhibition is direct or indirectly mediated by other anion channels. As expected, the formation of the low-diopter vacuoles was not blocked by the V-type H^+^ pump blocker BFA, whereas the formation of the bright vacuoles was completely blocked by BFA. The results further suggest that these low-diopter vacuoles are derived from the ER. Among the ER Ca^2+^ channel or Ca^2+^ pump blockers, tharpsigargin (TG) very significantly increased the sizes of both vacuoles (p < 0.01), and the IP3R blocker 2-APB decreased the sizes of both types of vacuoles (p < 0.05). The inhibition of the mitochondrion Ca^2+^ uniporter by RU360 only slightly decreased the size of both types of vacuoles (statistically not significant). These results imply that the cytosolic free Ca^2+^ level exerts an impact on the sizes of both types of vacuoles, because the TG treatment is able to increase the cytosolic free Ca^2+^ level, whereas 2-APB decreased the cytosolic free Ca^2+^ level by blocking the release of the Ca^2+^ stores through IP3R^29^,^30^,^31^. This evidence shows the Ca^2+^ sensitivity of Best3V2.

These results show that Best3V2 also induces ER swelling when it is confined to the ER, but Clc3s retention in the ER does not induce ER swelling. Amino acids 114–153 of Best3V2 comprise the key domain mediating its ability to bind lysosomes. The traditional anion channel blockers do not effectively block the formation of either the lysosome vacuoles or the ER vacuoles. However, both types of vacuoles display a Ca^2+^-dependent property.

In summary, Best3V2 is capable of exerting impacts on ER while Clc3s is not. Traditional anion channel blockers seem to be inefficiency in blocking both the vacuolization of ER and lysosome induced by Best3V2.

### Best3V2 reduces the free intracellular Ca^2+^ mobilization, which prevents premature excitation and contraction in differentiating myoblasts

**Figure 5:**
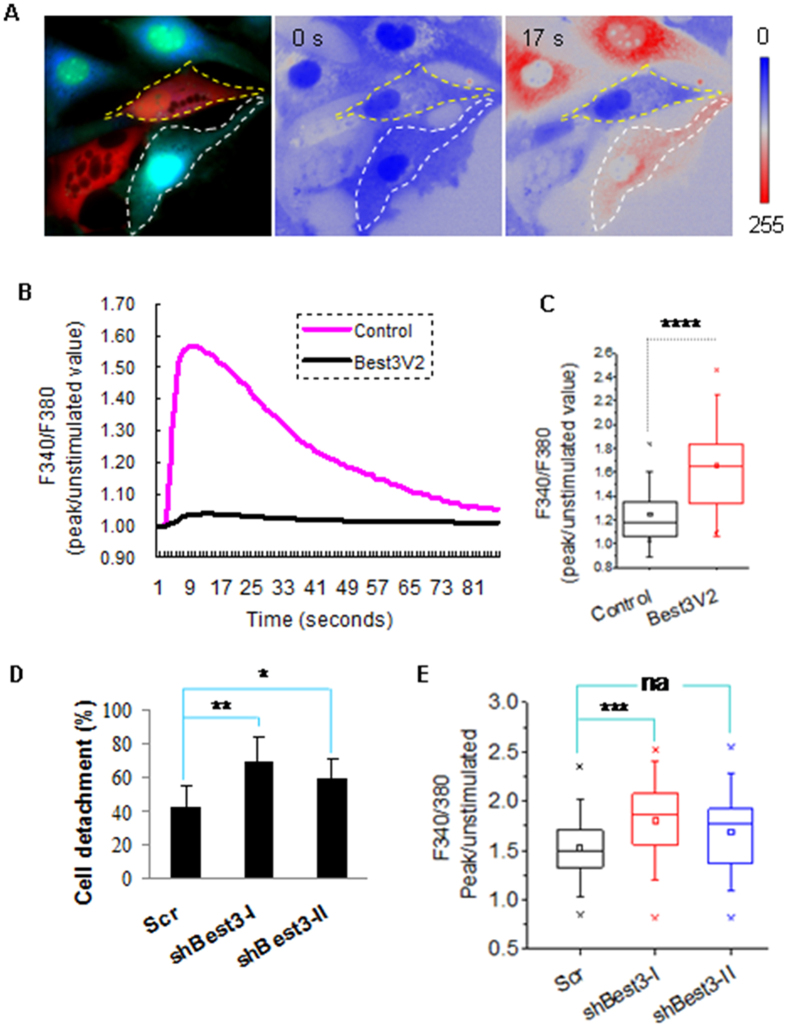
Best3V2 compromises calcium release from the intracellular Ca^2+^ stores, which inhibits myoblast contraction and detachment from the extracellular matrix. (**A**) Best3V2 overexpression inhibited ER Ca^2+^ release in NIH3T3 cells. The left panel was an IF image before bradykinin treatment and was shown in RGB color format. Blue denotes the fluorescence emission of Fura-2 excited at 340 nm, green denotes the fluorescence emission of Fura-2 excited at 380 nm, and red represents the transfected DsredN1. The color bar representing the intensity of the *F340*/*380* ratio was shown on the right. (**B**) Representative ratio (ratio of peak *F340*/*380* value against the *F340*/*380* value before stimulation) of the control cells and Best3V2-positive cells within the dashed cycles. (**C**) Box diagrams showed the average ratio (ratio of *F340*/*380* peak value against the *F340*/*380* value before stimulation) of the control cells and Best3V2-positive cells. Fifteen cells were randomly selected for counting. (**D**) Effect of knocking down endogenous Best3V2 expression on caffeine-induced C2C12 myoblast detachment from laminin-coated coverglasses. Ten cover glasses were counted for each treatment. *p < 0.05, **p < 0.01, ***p < 0.005, ****p < 0.001. (**E**) Effect of knocking down endogenous Best3V2 expression on ER Ca^2+^ release in C2C12 myoblasts. Twenty cells were randomly selected for counting. The experiments were repeated 3 times. All error bars denote the S.D. values.

We have shown that the ER can be altered by the retention of Best3V2 in this organelle. We then wondered whether Best3V2 without the ER retention signal impacted the ER ion balance, because BestV2 is synthesized in and transported by the ER. More importantly, Best3V2 was also shown to be specifically located in the ER around the nuclear envelope, as shown in Fig. 2D. Additionally, Best1 has been proposed to be able to alter ion homeostasis in the ER^32^,^33^,^34^,^35^. Therefore, we tested the alterations in ER Ca^2+^ release by overexpressing Best3V2 to determine whether the ER ion balance is altered. NIH3T3 cells were used for this experiment because these cells endogenously express the Best3 splice variants and thus have a more similar endogenous cell environment than HeLa cells. As expected, in the absence of the ER retention signal, we still detected a striking decrease in Best3V2-mediated ER Ca^2+^ release in bradykinin-stimulated NIH3T3 cells (Fig. 5A–C).

We then determined the role of endogenous Best3V2 in ER Ca^2+^ release. The expression of endogenous Best3V2 was knocked down in C2C12 myoblasts. To knockdown endogenous Best3, we constructed 2 shRNAs (shBest3-I and shBest3-II) against mouse Best3. The knockdown efficiency of shBest3-I was approximately 80% and that of shBest3-II was approximately 50%, respectively, which was validated by real-time PCR (data not shown). Unfortunately, it is difficult to get the myoblasts (infected with shBest3-I or shBest3-II) to attach to the laminin-coated coverglasses on day 5 postconfluency when they are treated with even low concentrations of caffeine (0.1 mM) to trigger ER Ca^2+^ release (Fig. 5D). In contrast, the Scr control was better at resisting detachment. We then tested whether this detachment was attributed to Ca^2+^-induced cell contraction. The peak Ca^2+^ release was significantly increased by shBest3-I upon caffeine stimulation (Fig. 5E). Endogenous Best3V2 may tune the amplitude of Ca^2+^ release from intracellular Ca^2+^ stores, such as from the perinuclear ER or lysosomes, which may prevent excessive fluctuations of the free Ca^2+^ concentrations in differentiating myoblasts. Unfortunately, we did not observe a visible alteration in C2C12 myoblast differentiation following Best3 knockdown, i.e., morphology and cell growth (data not shown). For differentiating myoblasts, we postulate that premature excitation and contraction may be objectively reduced by Best3V2 at certain differentiation stages. In addition, it may facilitate more stable attachment to the extracellular matrix during elongation. However, further detailed investigations are required to determine its exact physiological roles in myoblasts and mature muscles.

In summary, Best3V2 without the ER retention signal still exerts an impact on the ER in both NIH3T3 cells and C2C12 myoblasts. In C2C12 myoblasts, the endogenous Best3 splice variants inhibited excitation and contraction by regulating the cytosolic Ca^2+^ levels.

## Discussion

Our imaging data showed that Best3V2 alters the H^+^ gradient in lysosomes, which may be accompanied by alterations in the Ca^2+^ levels. These alterations impair lysosome-endosome hybrid reformation. For example, the luminal Ca^2+^ and V-type H^+^ ATPases have been suggested to be required for the condensation of the content and retrieval of components from endosome–lysosome hybrids^36^. Dysregulation of lysosomal channels results in the pathogenesis of many lysosome storage diseases^37^,^38^. Loss-of-function mutations in the lysosomal Cl^−^/H^+^ exchanger ClC-7 or its monotopic accessory protein Ostm1 result in osteopetrosis^39^,^40^. ClC-7 plays a prominent role in V-ATPase-mediated acid secretion^41^. Lysosomal Ca^2+^ dysfunction caused by an NPC1 mutation has been linked with human lysosome vacuolization^42^,^43^. Lysosome swelling has also been observed following the mutation of non-selective cation channel mucolipin-1 in lysosome, which results in a failure to reform lysosomes from hybrids and thereby causes the autosomal recessive disease mucolipidosis type IV, a lysosomal channelopathy leading to lipid storage^44^,^45^.

The ER and lysosome are the two main intracellular Ca^2+^ stores. The well-known NAADP-induced Ca^2+^ signals are from the lysosome Ca^2+^ store. The ER or SR is the major intracellular neutral Ca^2+^ store. The local Ca^2+^ signal from the plasma membrane or acidic Ca^2+^ stores are amplified by neighboring IP_3_ and RYRs on the ER by Ca^2+^-induced Ca^2+^ release (CICR) to mediate a larger global Ca^2+^ signal^46^,^47^. However, the Ca^2+^ release from the lysosome is also affected by the ER or SR. For example, the Ca^2+^ response to NAADP in the lysosome is suppressed by interfering with ER Ca^2+^ sequestration^48^. Therefore, the targeting of Best3V2 to both Ca^2+^ stores directly placed the 2 important intracellular Ca^2+^ stores under its control. Its physiological significance in myoblasts and muscles is still unknown. However, two putative consequences can be postulated. One is the inhibition of motility or contraction by reducing Ca^2+^ release from the SR. The other is the regulation of the Ca^2+^ levels within certain intracellular organelles. For the former, when mononuclear myoblasts merge into multinuclear myoblasts, attach to mature muscle fibers and elongate into long muscles, premature contraction may not be necessary for certain differentiation steps. For the latter, attention must be paid to determine how excess Ca^2+^ levels can be prevented in certain organelles, such as the nucleus, upon muscle contraction. Evidence shows that the nuclear calcium level is finely regulated and involves the outer nuclear envelope^49^,^50^,^51^. A considerable number of studies have shown that the same signals that regulate “excitation-contraction coupling” (EC-coupling) are also capable of regulating certain gene expression programs, a new paradigm coined “excitation-transcription coupling” (ET-coupling)^52^,^53^,^54^. Dysregulation of ET coupling led to problems with cell growth and gene expression. For example, in the adult heart, neurohormonal/mechanical stress enhances ET coupling, resulting in hypertrophy, the reexpression of the fetal gene program, and alterations in ion channel and transporter expression (see review^55^). In this context, the abundant distribution of Best3V2 on the nuclear envelope may form a barrier or cushion around the nuclei to reduce excess, local Ca^2+^ fluctuations upon excitation and contraction. Best3-deficient animal models are needed to verify this hypothesis. Additionally, the role of Best3V2 in muscle contraction (exercise intensity, duration, and frequency) should also be investigated further. *In vivo*, contraction may be finely regulated by strictly controlling the level of endogenous Best3V2. Under native conditions, Best3V2 promotes Ca^2+^ release from the ER at an appropriate level.

In the ER, the distribution of Ca^2+^ channels in the ER membrane is discontinuous, with clustering in certain locations and different Ca^2+^ concentrations in sub-compartments^56^,^57^,^58^. However, the ER luminal Ca^2+^ movements show that this organelle system is one continuous Ca^2+^ store with a Ca^2+^ tunnel effect^59^,^60^. The location of Best3V2 on the nuclear envelope may alter the Ca^2+^ levels in the adjacent ER or even throughout the ER.

However, our imaging data imply that the Best3V2-induced phenomena in the lysosome and ER cannot be explained by Best3V2 having a role as an anionic channel. For example, because Best3V2 works as an anion channel, Best3V2 overexpression should theoretically increase the H^+^ levels in the lysosome and Ca^2+^ levels in the ER, which in turn will render the lysosome more acidic and initiate higher peak Ca^2+^ release in the ER. Our results show the opposite trend. This observation can be explained by 2 possible roles for Best3V2. One explanation is that, unlike full-length Best3, Best3V2 works as a cation channel, which is similar to the cation conductance of the bacterial Bestrophin. The other possible explanation is that Best3V2 loses channel activity, but it indirectly influences other cation channels in the lysosomes and ER. However, additional studies are required to characterize its ion conductance within intracellular organelles or in artificial lipid bilayers.

In conclusion, this manuscript provides evidence that a rare splice variant of Best3 targets both the lysosome and the ER around the nuclear envelope. The splice variant is mainly expressed in myoblasts and muscles, and functions to regulate Ca^2+^ release from cytosolic stores. Its inhibition of Ca^2+^ release may help prevent premature contraction in myoblasts or prevent Ca^2+^ overloading to the nucleus in mature muscles. However, its exact physiological roles *in vivo* require further verification in animal models. The expression of Best3V2 in myoblasts and muscle cells suggests that the Ca^2+^ regulation in these cells is complicated, and additional novel channels are involved in its fine control.

## Materials and Methods

### Reagents, Materials and cells

Dulbecco’s Modified Eagle’s Medium (DMEM), glutamine, trypsin-EDTA, Penicillin/streptomycin, fetal bovine serum (FBS), Lysosensor DND-160 and Fura-2-AM were obtained from Invitrogen (Shanghai, China). The pAbs (anti-LC3B and anti-GST tag), mAbs (anti-HA, anti-Flag tag and anti-Actin IgG), and anti-tropomyosin mAb were purchased from Sigma-Aldrich (Shanghai, China); the anti-Best3V2 pAb (sc-70147), anti-Caveolin1 pAb (sc-894) and anti-Rab7 mAb (sc-376362) were purchased from Santa Cruz Biotechnology (CA, USA). The anti-HA mAb (rabbit) (AP1012a), anti-cathepsin D mAb (CTSD) (AM2221b) and anti-ATG7 mAb (AP1813d) were obtained from Abgent (Wuxi, China). The anti-Best3 pAb (20443-1-AP) was obtained from Proteintech (Wuhan, China). The anti-P62 pAb (PM045) was purchased from MBL (Aichi, JAPAN). The anti-PDI mAb, anti-GAPDH mAb, anti-GFP pAb and anti-ATG7 pAb were purchased from Beyotime (Shanghai, China). The HRP-conjugated secondary antibodies were obtained from Abgent (Wuxi, China). The Alexa Fluor 488- and Alexa Fluor 555-conjugated secondary antibodies were obtained from Invitrogen. The anti-Flag M2 magnetic beads were purchased from Sigma-Aldrich. Nocodazole, bafilomycin A1, bradykinin, 5-nitro-2-(3-phenylpropylamino) benzoic acid (NPPB), niflumic acid (NFA), 4′-diisothiocyanatostilbene-2,2′-disulfonic acid disodium salt hydrate (DIDS), thapsigargin (TG), 2-aminoethoxydiphenyl borate (2-APB) and acridine orange (AO) were obtained from Sigma-Aldrich. RU360 was purchased from Calbiochem (Merck Millipore, Beijing, China). 3-Methyladenine (3-MA), Wortmannin and LY294002 were purchased from Selleckchem (TX, USA). The restriction enzymes were obtained from Thermo Scientific (Fermentas, Thermo Scientific, Shanghai, China) and New England Biolabs (MA, USA). The Turbofect transfection reagent was obtained from Fermentas and Lipofectamine 2000 was purchased from Invitrogen. Ni-NTA Superflow FF was obtained from GE Healthcare (Shanghai, China). The PIP strips (p-6001) were purchased from Echelon Biosciences (UT, USA). All other chemicals were from Sigma-Aldrich and Sangon Biotech (Shanghai, China). The HeLa, NIH3T3 and C2C12 cells were purchased from ATCC. All experiments were performed according to the guidelines of the Committee of Qingdao Agricultural University.

### RT-PCR, DNA constructs and primers

Total RNAs were isolated with Trizol (Invitrogen). Hiscript II transcriptase (Vazyme, Nanjing, China) was used to synthesize the cDNAs. Three micrograms of the total RNAs from muscle tissues or cell lines were transcribed into cDNAs. Splice variants V1 and V2 of Best3 were cloned from C2C12 myoblasts via RT-PCR. The GFP_LC3a, GFP_FAK, Clc-3 short isoform (Clc3s), Rab7, Rab5, DNM2, CDC42, and VPS4 sequences were cloned from 293T cells into a modified pcDNA3.1^+^ vector (Invitrogen) with an HA tag or pEGFPC1 via RT-PCR. The dominant negative mutants Rab5a (S34N), Dnm2 (K44A), VPS4 (E228Q), Rab7 (T22N), Rab7 (N125I), and CDC42 (T17N) and the constitutively active Rab7 (Q67L) mutant was constructed by PCR.

The plasmids encoding mouse Bestrophin 3 were kindly provided by Dr. HC Hartzell (Emory University School of Medicine, USA). All truncated Best3 constructs and mutations were generated by PCR and then subcloned into modified pcDNA3.1^+^ vectors (Invitrogen) containing C-terminal HA, Flag or GST tags, which were created in our lab, or into pEGFP vectors with a C-terminal GFP tag (Clontech, Takara Bio., CA, USA). The shRNA vector pLKO.1-TRC2 (Plasmid #10878) and lentivirus packaging plasmids psPAX2 (Plasmid #12260) and pMD2.G (Plasmid #12259) were obtained from Addgene (MA, USA). All of the resulting constructs were validated by sequencing. The primers for Best3 RT-PCR were: 465-1125 primer pairs, forward 5′-GACGGCAGATCGTTTGAAGATTTCT-3′, Reverse: 5′-GGTCCATGGTGGGAAACCTCTTG-3′; and 1028-1515 primer pairs, forward: 5′-CGACCACCTCAAATCGCCT-3′, reverse: 5′-tcttcatcttgggcaaactcat-3′.

### Fluorescence Microscopy

For the immunofluorescence analysis, the cells were plated onto coverslips coated with 0.1% gelatin (Sigma Aldrich) in 12-well plates and fixed with 3% paraformaldehyde (PFA) (Songon, Shanghai) at room temperature. The fixed cells were permeabilized with cold ethanol, phosphate-buffered saline (PBS) containing 0.2% Triton X-100, or PBS containing 0.5% saponin. Nonspecific binding sites were blocked by incubating the coverslips with 3% BSA for 20 min. The fixed cells were incubated with primary antibodies for 1–2 h and with the appropriate Alexa Fluor-conjugated secondary antibodies for 1 h before mounting. The slides were visualized under a confocal or fluorescence microscope. The images were further analyzed with the FV10-ASW 3.0 viewer software.

For live cell imaging, the cells were grown in 35-mm glass-bottom dishes (MatTek or Nest) and staged into a heated incubation chamber with CO_2_. The cells were imaged using an Olympus FV-10 confocal microscope or a fluorescent microscope, and the images were acquired with the FV10-ASW 3.0 viewer (Olympus) or Metamorph (Molecular Devices) software. The image stacks or movie files were further processed with ImageJ software. A phase microscope was used for vacuole imaging and counting. The vacuole sizes were calculated with blind method in digitally enlarged images on a computer screen using a scale ruler.

### Transmission Electron Microscopy

For the ultrastructural analysis of the vacuoles, the cells were fixed in 1% (vol/vol) PFA/2.5% (vol/vol) glutaraldehyde in 100 mM PBS, pH 7.2, for 3 days at 4 °C. The samples were washed in PBS and post fixed in 0.5% (wt/vol) osmium tetroxide/0.08% (wt/vol) potassium ferricyanide/100 mM PBS for 1 h, followed by 1% (vol/vol) tannic acid/100 mM PBS for 1 h. The samples were then rinsed in distilled H_2_O before staining with 1% (vol/vol) aqueous uranyl acetate for 1 h. Following several rinses in distilled H_2_O, the samples were dehydrated in a graded series of ethanol and embedded in resin. Sections (95 nm) were cut and lifted onto copper grids and stored overnight on gelatin at 4 °C. The sections then were stained with lead citrate and uranyl acetate, and viewed on a transmission electron microscope (H-7500; Hitachi).

### Acridine orange (AO) staining and Oil Red O staining

Live cells seeded in glass-bottom dishes (Nest Biotechnology) were stained with AO (Sigma Aldrich) (5 μg/ml) in DMEM medium for 15 min. The cells were washed 3 times with PBS and visualized under microscope. For Oil red O staining, the cells were fixed with 4% paraformaldehyde for 40 min at room temperature. Following several rinses in PBS and distilled H_2_O, the cells were treated with 60% isopropanol for 3 min. Then, an Oil Red O Solution (0.36% Oil Red O in 60% isopropanol) (Sigma-Aldrich) was added, and the cells were stained at room temperature for 50 min. The cells were rinsed 3 times with water and visualized under an AMEX-1200 microscope (AMG).

### Recombinant proteins and PIP strip blotting

Recombinant His_6_-HA tagged mouse Best 3 proteins, aa 114–153 and aa 114–153 (R125Q, R126Q), were expressed in the BL_21_ bacterial strain and then purified with Ni-NTA Superflow FF (GE Healthcare) according to the manufacturer’s instructions. The PIP strips (Echelon Biosciences) were incubated with 0.05 μg/ml recombinant proteins for 30 min in 2% BSA-PBS and then incubated with an anti-HA tag antibody for 30 min and an HRP-conjugated secondary antibody for 30 min. The strips were developed with X-ray film.

### Biotinylation of the proteins on the plasma membrane surface

Plasma membrane targeting was monitored via cell surface protein biotinylation and isolation with Cell Surface Protein Isolation Kit (Pierce, Thermo Fisher Scientific, USA), according to the manufacturer’s instructions. Four days after the cells reached confluency, the C2C12 cells were exposed to membrane-impermeable Sulfo-NHS-LC biotin for 30 min on ice. The biotinylated membrane proteins were solubilized and affinity-purified with streptavidin beads. The purified proteins were subjected to Western blotting with the Best3 antibody, followed by the horseradish peroxidase-conjugated secondary antibody. The protein concentration was assayed by the Bradford protein assay and biotinylated membrane proteins for myoblat and mature skeletal muscles were equally loaded for Western blotting.

### Cell lysis and Western blots

For the immunoblot, the cells were placed on ice, washed twice in cold PBS, and lysed in mild RIPA buffer [50 mM Tris, pH 7.4, 150 mM NaCl, 0.5 mM DTT, 2mM EDTA, and 1% NP-40] with protease inhibitors. The Western blots were performed by separating the samples on SDS-PAGE gels, which were transferred to PVDF membrane for blotting. The concentration was assayed by the Bradford protein assay method.

### Satellite cell isolation from mouse skeletal muscles

C57BL/6 mice were purchased from Vital River Laboratories (Beijing, China). All experimental protocols were approved by the Institutional Animal Care and Use Committee of Qingdao Agricultural University. All experiments were performed according to the guidelines of the Institutional Animal Care and Use Committee of Qingdao Agricultural University and Tianjin University of Science and Technology. Muscle tissues from 6-week-old mice were minced into small pieces and incubated in MEM with 10% FBS and 0.2% collagenase (type I, Sigma) at 37 °C in a water-saturated incubator with 5% CO_2_ for approximately 2.5 h. The muscles were transferred with wide-mouth Pasteur pipette into MEM, teased with forceps, and triturated to release single fibers. The cells were cultured in MEM with 10% FBS, and the medium was changed every 3 days. Approximately 10–20 single fibers per well were grown on laminin-coated cover glasses in a 12-well plate. The bottom of the 35-mm plate or cover glass was coated with 10 μg/ml of laminin (Invitrogen) at 37 °C for 2 h. The laminin coating solution was removed immediately before the myocytes were plated.

### ER Ca^2+^ release assay

NIH3T3 cells were seeded at low density on glass coverslips and used the following day for the ratiometric imaging measurements of Ca^2+^ release from the ER. The cells were loaded in tissue culture medium with fura-2-AM (5 μM) for 20–30 min at room temperature. The experiments were performed in Ringer’s solution containing (in mM) 121 NaCl, 2.4 K_2_HPO_4_, 0.4 KH_2_PO_4_, 1.2 CaCl_2_, 1.2 MgCl_2_, 5.5 glucose, and 10 Hepes/NaOH, pH 7.2. The cells were continuously superfused on a heated microscope stage in an open Leiden chamber. Coverslips with dye-loaded cells were mounted into a heated metal flow-through perfusion chamber placed on the stage of an inverted Olympus fluorescence microscope. A one-half volume of bradykinin in Ringer’s solution was added to the chamber to a final concentration of 10 nM with a pipette within seconds. The fluorescence emitted from the cells was measured in response to alternating excitation at 340 and 380 nm using a computer-controlled four-place sliding filter holder. Time lapse images were recorded at 1 s intervals after stimulation with 10 nM bradykinin. The images at 0 s and 17 s were shown. DsredN1 was co-transfected with Best3V2 at a 1:3 ratio as an indicator of the positively transfected cells. All measurements were automatically corrected for background. The ratio of light emitted from fura-2 at the two excitation wavelengths (340/380) provides a measure of the ionized cytoplasmic Ca^2+^ concentrations.

For the C2C12 myoblast assay, C2C12 cells were grown in DMEM containing 10% FBS and maintained in a humidified incubator at 37 °C in the presence of 5% CO_2_/95% air until the cells were completely confluent, which was designated day 0. Then, the cells were transferred to DMEM with 1% horse serum to initiate differentiation into myoblasts. For lentivirus infection, virus particles were added before the cells reached confluency to obtain a better infection. The cells were also treated with puromycin to select the positively infected cells 24 hours after infection. On day 2 after confluency, the myoblasts were assayed in the intracellular Ca^2+^ release assay. Fura 2-AM was applied to the C2C12 myoblasts for the Ca^2+^ release assay, similar to the NIH3T3 cells. The images were further analyzed using ImageJ software.

### shRNAi

For shRNAi manipulation, the HeLa cells were infected with a lentivirus encoding shRab7a or Scramble (Scr) for 2 days. Then, the cells were treated with 1 μg/ml puromycin for 24 h after infection, and then these cells were further transfected with Best3V2_HA or Clc3s, respectively. For RNAi in the C2C12 myoblasts, the C2C12 cells were seeded on laminin-coated coverslips and infected with shBest3-I, shBest3-II, or scrambled control lentiviruses. Validated lentiviral hairpin constructs of shRab7a were obtained from Sigma-Aldrich.

The sequence targeting shRab7a was: 5′-GGCTAGTCACAATGCAGATAT-3′.

The sequence targeting shBest3-I was: 5′-GACAGGCTGATGCTCCTCATT-3′.

The sequence targeting shBest3-II was: 5′-GCAGATGAGAGGAAATTATTC-3′.

### Blocking of ion channels or ion pumps

Bafilomycin (BFA): Twelve hours after Best3V2_HA transfection, HeLa cells were treated with BFA (2 nM) for 12 h.

Anion channel blockers NPPB, DIDS and NFA: HeLa cells were transfected with Best3V2 for 12 h and then treated with the following anion channel blockers: 50 μM NPPB, 100 μM NFA, and 100 μM DIDS. DMSO was added to the controls at a final concentration of 0.2%.

TG, 2-APB and RU360: The cells were treated with 2 μM Tharpsigargin (TG, non-competitive inhibitor of intracellular Ca^2+^ pumps (SERCA)), 30 μM 2-APB (an antagonist of the IP3 receptor and TRP channel) or 10 μM RU360 (mitochondrial calcium uptake blocker) for 12 h.

### Cell detachment assay of differentiating C2C12

The C2C12 cells were seeded on laminin-coated coverslips and infected with a lentivirus before they reached confluency. On day 5 after confluency, C2C12 myoblasts grown on glass coverslips were subjected to a detachment assay by treating them with caffeine (0.1 mM) for 10 min at 37 °C. The long-shaped cells were usually bound together to form a thin cell layer on the coverslips. When the cells detached, the cells still bound together very closely, and thus the thin cell layer can be seen by eye. The number of coverslips with a detached cell layer was calculated.

### Statistical analysis

Statistically significant differences were determined using the two-tailed Student’s t-test in Excel. Where applicable, the data were expressed as the means + S.D. Unless indicated otherwise, the experiments were repeated 3 times.

## Additional Information

**How to cite this article**: Wu, L. *et al.* A C-terminally truncated mouse Best3 splice variant targets and alters the ion balance in lysosome-endosome hybrids and the endoplasmic reticulum. *Sci. Rep.* **6**, 27332; doi: 10.1038/srep27332 (2016).

## Supplementary Material

Supplementary Information

## References

[b1] Sun H., Tsunenari T., Yau K. W. & Nathans J. The vitelliform macular dystrophy protein defines a new family of chloride channels. Proc Natl Acad Sci USA 99, 4008–4013, doi: 10.1073/pnas.052692999 (2002). 11904445 PMC122639

[b2] Qu Z., Wei R. W., Mann W. & Hartzell H. C. Two bestrophins cloned from Xenopus laevis oocytes express Ca(2+)-activated Cl(−) currents. J Biol Chem 278, 49563–49572, doi: 10.1074/jbc.M308414200 (2003). 12939260

[b3] Qu Z., Fischmeister R. & Hartzell C. Mouse bestrophin-2 is a bona fide Cl(−) channel: identification of a residue important in anion binding and conduction. J Gen Physiol 123, 327–340, doi: 10.1085/jgp.200409031 (2004). 15051805 PMC2217464

[b4] Tsunenari T. *et al.* Structure-function analysis of the bestrophin family of anion channels. J Biol Chem 278, 41114–41125, doi: 10.1074/jbc.M306150200 (2003). 12907679 PMC2885917

[b5] Yang Y. D. *et al.* TMEM16A confers receptor-activated calcium-dependent chloride conductance. Nature 455, 1210–1215, doi: 10.1038/nature07313 (2008). 18724360

[b6] Caputo A. *et al.* TMEM16A, a membrane protein associated with calcium-dependent chloride channel activity. Science 322, 590–594, doi: 10.1126/science.1163518 (2008). 18772398

[b7] Fischmeister R. & Hartzell H. C. Volume sensitivity of the bestrophin family of chloride channels. J Physiol 562, 477–491, doi: 10.1113/jphysiol.2004.075622 (2005). 15564283 PMC1665509

[b8] Chien L. T. & Hartzell H. C. Drosophila bestrophin-1 chloride current is dually regulated by calcium and cell volume. J Gen Physiol 130, 513–524, doi: 10.1085/jgp.200709795 (2007). 17968025 PMC2151665

[b9] Qu Z. & Hartzell H. C. Bestrophin Cl^−^ channels are highly permeable to HCO_3_. Am J Physiol Cell Physiol 294, C1371–1377, doi: 10.1152/ajpcell.00398.2007 (2008). 18400985 PMC2465210

[b10] Yu K., Lujan R., Marmorstein A., Gabriel S. & Hartzell H. C. Bestrophin-2 mediates bicarbonate transport by goblet cells in mouse colon. J Clin Invest 120, 1722–1735, doi: 10.1172/JCI41129 (2010). 20407206 PMC2860923

[b11] Lee S. *et al.* Channel-mediated tonic GABA release from glia. Science 330, 790–796, doi: 10.1126/science.1184334 (2010). 20929730

[b12] Kane Dickson V., Pedi L. & Long S. B. Structure and insights into the function of a Ca(2+)-activated Cl(−) channel. Nature 516, 213–218, doi: 10.1038/nature13913 (2014). 25337878 PMC4454446

[b13] Yang T. *et al.* Structure and selectivity in bestrophin ion channels. Science 346, 355–359, doi: 10.1126/science.1259723 (2014). 25324390 PMC4341822

[b14] Hartzell H. C., Qu Z., Yu K., Xiao Q. & Chien L. T. Molecular physiology of bestrophins: multifunctional membrane proteins linked to best disease and other retinopathies. Physiol Rev 88, 639–672, doi: 10.1152/physrev.00022.2007 (2008). 18391176

[b15] Marquardt A. *et al.* Mutations in a novel gene, VMD2, encoding a protein of unknown properties cause juvenile-onset vitelliform macular dystrophy (Best’s disease). Hum Mol Genet 7, 1517–1525 (1998). 9700209 10.1093/hmg/7.9.1517

[b16] Petrukhin K. *et al.* Identification of the gene responsible for Best macular dystrophy. Nature genetics 19, 241–247, doi: 10.1038/915 (1998). 9662395

[b17] Zhang Y. *et al.* Suppression of Ca^2+^ signaling in a mouse model of Best disease. Hum Mol Genet 19, 1108–1118, doi: 10.1093/hmg/ddp583 (2010). 20053664 PMC2830833

[b18] Bakall B. *et al.* Bestrophin-2 is involved in the generation of intraocular pressure. Invest Ophthalmol Vis Sci 49, 1563–1570, doi: 10.1167/iovs.07-1338 (2008). 18385076 PMC2832837

[b19] Zhang Y. *et al.* Enhanced inflow and outflow rates despite lower IOP in bestrophin-2-deficient mice. Invest Ophthalmol Vis Sci 50, 765–770, doi: 10.1167/iovs.08-2501 (2009). 18936135 PMC2840997

[b20] Kramer F., Stohr H. & Weber B. H. Cloning and characterization of the murine Vmd2 RFP-TM gene family. Cytogenet Genome Res 105, 107–114, doi: 10.1159/000078016 (2004). 15218265

[b21] Luzio J. P., Pryor P. R. & Bright N. A. Lysosomes: fusion and function. Nat Rev Mol Cell Biol 8, 622–632, doi: 10.1038/nrm2217 (2007). 17637737

[b22] Hyttinen J. M., Niittykoski M., Salminen A. & Kaarniranta K. Maturation of autophagosomes and endosomes: a key role for Rab7. Biochim Biophys Acta 1833, 503–510, doi: 10.1016/j.bbamcr.2012.11.018 (2013). 23220125

[b23] Kummel D. & Ungermann C. Principles of membrane tethering and fusion in endosome and lysosome biogenesis. Curr Opin Cell Biol 29, 61–66, doi: 10.1016/j.ceb.2014.04.007 (2014). 24813801

[b24] Roth M. G. Phosphoinositides in constitutive membrane traffic. Physiol Rev 84, 699–730, doi: 10.1152/physrev.00033.2003 (2004). 15269334

[b25] Li X., Wang T., Zhao Z. & Weinman S. A. The ClC-3 chloride channel promotes acidification of lysosomes in CHO-K1 and Huh-7 cells. Am J Physiol Cell Physiol 282, C1483–1491, doi: 10.1152/ajpcell.00504.2001 (2002). 11997263

[b26] Jackson M. R., Nilsson T. & Peterson P. A. Identification of a consensus motif for retention of transmembrane proteins in the endoplasmic reticulum. EMBO J 9, 3153–3162 (1990). 2120038 10.1002/j.1460-2075.1990.tb07513.x PMC552044

[b27] Munro S. & Pelham H. R. A C-terminal signal prevents secretion of luminal ER proteins. Cell 48, 899–907 (1987). 3545499 10.1016/0092-8674(87)90086-9

[b28] Milenkovic V. M., Rivera A., Horling F. & Weber B. H. Insertion and topology of normal and mutant bestrophin-1 in the endoplasmic reticulum membrane. J Biol Chem 282, 1313–1321, doi: 10.1074/jbc.M607383200 (2007). 17110374

[b29] Jackson T. R., Patterson S. I., Thastrup O. & Hanley M. R. A novel tumour promoter, thapsigargin, transiently increases cytoplasmic free Ca^2+^ without generation of inositol phosphates in NG115-401L neuronal cells. Biochem J 253, 81–86 (1988). 3138987 10.1042/bj2530081 PMC1149260

[b30] Ma H. T. *et al.* Requirement of the inositol trisphosphate receptor for activation of store-operated Ca^2+^ channels. Science 287, 1647–1651 (2000). 10698739 10.1126/science.287.5458.1647

[b31] Thastrup O., Cullen P. J., Drobak B. K., Hanley M. R. & Dawson A. P. Thapsigargin, a tumor promoter, discharges intracellular Ca^2+^ stores by specific inhibition of the endoplasmic reticulum Ca2(+)-ATPase. Proc Natl Acad Sci USA 87, 2466–2470 (1990). 2138778 10.1073/pnas.87.7.2466 PMC53710

[b32] Barro-Soria R. *et al.* ER-localized bestrophin 1 activates Ca^2+^-dependent ion channels TMEM16A and SK4 possibly by acting as a counterion channel. Pflugers Arch 459, 485–497, doi: 10.1007/s00424-009-0745-0 (2010). 19823864

[b33] Gomez N. M., Tamm E. R. & Straubeta O. Role of bestrophin-1 in store-operated calcium entry in retinal pigment epithelium. Pflugers Arch 465, 481–495, doi: 10.1007/s00424-012-1181-0 (2013). 23207577

[b34] Neussert R., Muller C., Milenkovic V. M. & Strauss O. The presence of bestrophin-1 modulates the Ca^2+^ recruitment from Ca^2+^ stores in the ER. Pflugers Arch 460, 163–175, doi: 10.1007/s00424-010-0840-2 (2010). 20411394

[b35] Strauss O., Neussert R., Muller C. & Milenkovic V. M. A potential cytosolic function of bestrophin-1. Adv Exp Med Biol 723, 603–610, doi: 10.1007/978-1-4614-0631-0_77 (2012). 22183384

[b36] Pryor P. R., Mullock B. M., Bright N. A., Gray S. R. & Luzio J. P. The role of intraorganellar Ca(2+) in late endosome-lysosome heterotypic fusion and in the reformation of lysosomes from hybrid organelles. J Cell Biol 149, 1053–1062 (2000). 10831609 10.1083/jcb.149.5.1053 PMC2174832

[b37] Ruivo R., Anne C., Sagne C. & Gasnier B. Molecular and cellular basis of lysosomal transmembrane protein dysfunction. Biochim Biophys Acta 1793, 636–649, doi: 10.1016/j.bbamcr.2008.12.008 (2009). 19146888

[b38] Xu H. & Ren D. Lysosomal physiology. Annu Rev Physiol 77, 57–80, doi: 10.1146/annurev-physiol-021014-071649 (2015). 25668017 PMC4524569

[b39] Frattini A. *et al.* Chloride channel ClCN7 mutations are responsible for severe recessive, dominant, and intermediate osteopetrosis. J Bone Miner Res 18, 1740–1747, doi: 10.1359/jbmr.2003.18.10.1740 (2003). 14584882

[b40] Steward C. G. Neurological aspects of osteopetrosis. Neuropathol Appl Neurobiol 29, 87–97 (2003). 12662317 10.1046/j.1365-2990.2003.00474.x

[b41] Kornak U. *et al.* Loss of the ClC-7 chloride channel leads to osteopetrosis in mice and man. Cell 104, 205–215 (2001). 11207362 10.1016/s0092-8674(01)00206-9

[b42] Lloyd-Evans E. *et al.* Niemann-Pick disease type C1 is a sphingosine storage disease that causes deregulation of lysosomal calcium. Nat Med 14, 1247–1255, doi: 10.1038/nm.1876 (2008). 18953351

[b43] Lloyd-Evans E. & Platt F. M. Lysosomal Ca(2+) homeostasis: role in pathogenesis of lysosomal storage diseases. Cell Calcium 50, 200–205, doi: 10.1016/j.ceca.2011.03.010 (2011). 21724254

[b44] Piper R. C. & Luzio J. P. CUPpling calcium to lysosomal biogenesis. Trends Cell Biol 14, 471–473, doi: 10.1016/j.tcb.2004.07.010 (2004). 15350973

[b45] Remis N. N. *et al.* Mucolipin co-deficiency causes accelerated endolysosomal vacuolation of enterocytes and failure-to-thrive from birth to weaning. PLoS genetics 10, e1004833, doi: 10.1371/journal.pgen.1004833 (2014). 25521295 PMC4270466

[b46] Kamishima T. & Quayle J. M. Ca^2+^-induced Ca^2+^ release in cardiac and smooth muscle cells. Biochem Soc Trans 31, 943–946, doi: 10.1042/ (2003) . 14505454 10.1042/bst0310943

[b47] Petersen O. H. Ca signalling in the endoplasmic reticulum/secretory granule microdomain. Cell Calcium, doi: 10.1016/j.ceca.2015.01.006 (2015). 25662795

[b48] Ronco V. *et al.* A novel Ca(2)(+)-mediated cross-talk between endoplasmic reticulum and acidic organelles: implications for NAADP-dependent Ca(2)(+) signalling. Cell Calcium 57, 89–100, doi: 10.1016/j.ceca.2015.01.001 (2015). 25655285

[b49] Abrenica B. & Gilchrist J. S. Nucleoplasmic Ca(2+) loading is regulated by mobilization of perinuclear Ca(2+). Cell Calcium 28, 127–136, doi: 10.1054/ceca.2000.0137 (2000). 10970769

[b50] Gerasimenko O. V., Gerasimenko J. V., Tepikin A. V. & Petersen O. H. ATP-dependent accumulation and inositol trisphosphate- or cyclic ADP-ribose-mediated release of Ca^2+^ from the nuclear envelope. Cell 80, 439–444 (1995). 7859285 10.1016/0092-8674(95)90494-8

[b51] Malviya A. N. & Klein C. Mechanism regulating nuclear calcium signaling. Can J Physiol Pharmacol 84, 403–422, doi: 10.1139/y05-130 (2006). 16902586

[b52] Anderson M. E. Connections count: excitation-contraction meets excitation-transcription coupling. Circ Res 86, 717–719 (2000). 10764402 10.1161/01.res.86.7.717

[b53] Wamhoff B. R., Bowles D. K. & Owens G. K. Excitation-transcription coupling in arterial smooth muscle. Circ Res 98, 868–878, doi: 10.1161/01.RES.0000216596.73005.3c (2006). 16614312

[b54] Dominguez-Rodriguez A., Ruiz-Hurtado G., Benitah J. P. & Gomez A. M. The other side of cardiac Ca(2+) signaling: transcriptional control. Front physiol 3, 452, doi: 10.3389/fphys.2012.00452 (2012). 23226134 PMC3508405

[b55] Heineke J. & Molkentin J. D. Regulation of cardiac hypertrophy by intracellular signalling pathways. Nat Rev Mol Cell Biol 7, 589–600, doi: 10.1038/nrm1983 (2006). 16936699

[b56] Golovina V. A. & Blaustein M. P. Spatially and functionally distinct Ca^2+^ stores in sarcoplasmic and endoplasmic reticulum. Science 275, 1643–1648 (1997). 9054358 10.1126/science.275.5306.1643

[b57] Montero M. *et al.* Ca^2+^ homeostasis in the endoplasmic reticulum: coexistence of high and low [Ca^2+^] subcompartments in intact HeLa cells. J Cell Biol 139, 601–611 (1997). 9348278 10.1083/jcb.139.3.601 PMC2141710

[b58] Petersen O. H., Tepikin A. & Park M. K. The endoplasmic reticulum: one continuous or several separate Ca(2+) stores? Trends Neurosci 24, 271–276 (2001). 11311379 10.1016/s0166-2236(00)01787-2

[b59] Subramanian K. & Meyer T. Calcium-induced restructuring of nuclear envelope and endoplasmic reticulum calcium stores. Cell 89, 963–971 (1997). 9200614 10.1016/s0092-8674(00)80281-0

[b60] Park M. K., Petersen O. H. & Tepikin A. V. The endoplasmic reticulum as one continuous Ca(2+) pool: visualization of rapid Ca(2+) movements and equilibration. EMBO J 19, 5729–5739, doi: 10.1093/emboj/19.21.5729 (2000). 11060024 PMC305795

