# Supplementary material for: A C-terminally truncated mouse Best3 splice variant targets and alters the ion balance in lysosome-endosome hybrids and the endoplasmic reticulum
Source: Sci Rep. 2016 Jun 6;6:27332. doi: 10.1038/srep27332 (PMC4893618; doi:10.1038/srep27332)
Supplement: Supplementary Information [file srep27332-s1.pdf]

A C-terminus truncated mouse Best3 splice variant targets and alters the ion balance of lysosome-endosome hybrids and the endoplasmic reticulum

Lichang Wu, Yu Sun, Liqiao Ma, Jun Zhu, Baoxia Zhang, Qingjie Pan, Yuyin Li, Huanqi Liu, Aipo Diao, Yinchuan Li

Supplementary figures and figure legends

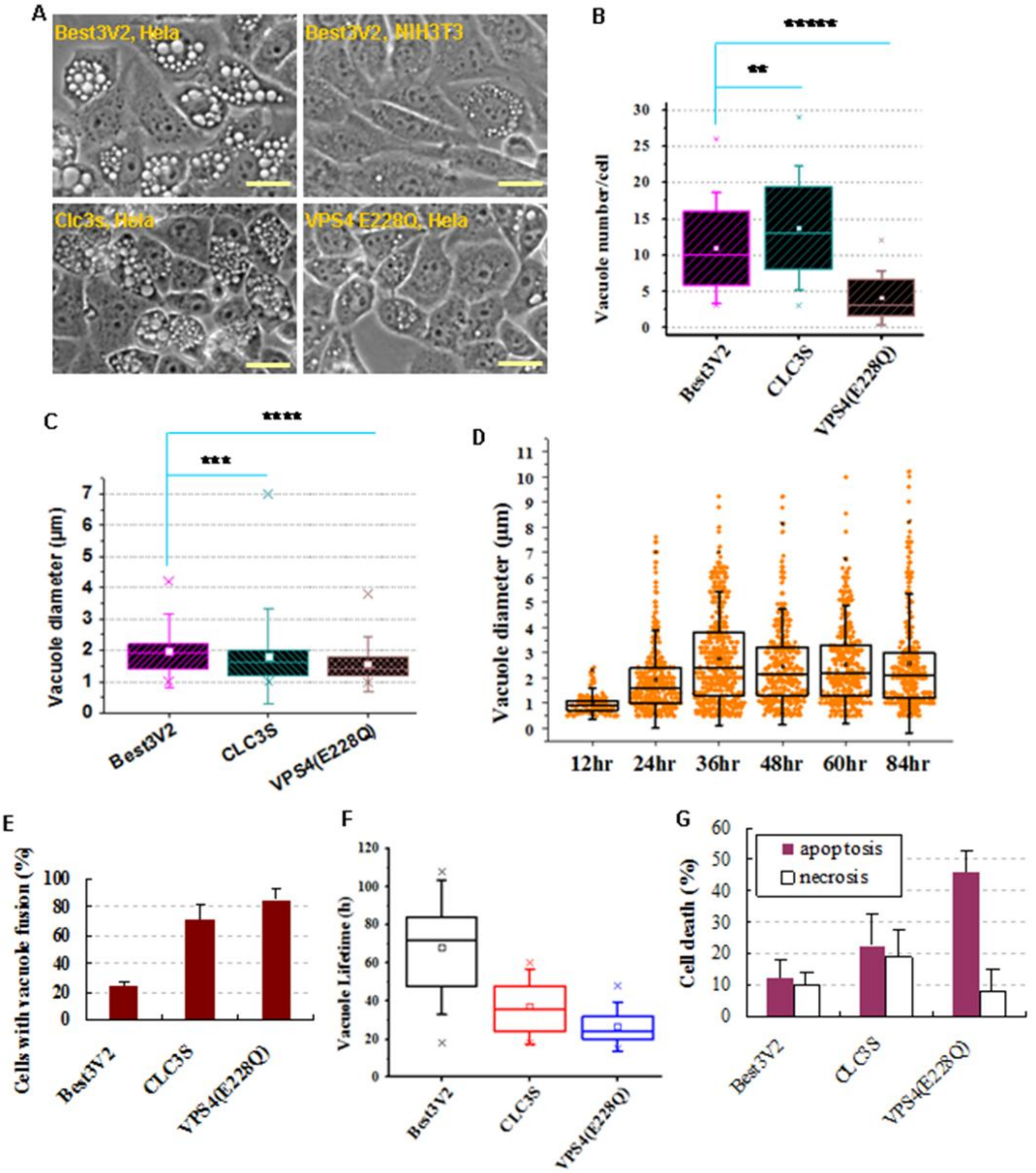

Figure S1: Distinct properties of vacuoles induced by Best3-V2, Clc3s or VPS4(E228Q) (A) Morphology of vacuoles induced by Best3-V2_HA in Hela cells and NIH3T3 cells, compared with the vacuoles induced by Clc3 short natural splice variant form (Clc3s, without N-terminal 58 aa) and vacuoles induced by VPS4 dominant negative mutant (VPS4(E228Q)) in Hela cells. (B) Scatter and box diagrams showed the average vacuole number per cell induced by Best3V2, Clc3s and VPS4(E228Q). 50 cells with vacuoles were randomly selected for counting. Error bars denote S.D. X, percentage from 1% to 99%. Two boxes represent 25% and 75% percent of all vacuoles respectively. Small rectangle within box denotes the average value. All following box and scatter diagrams below are the same setting without further indication. Cells used below are Hela cells

unless indicated other cells. We indicate vacuoles induced by Best3V2_HA as Vac-V2, vacuoles induced by Clc3s as Vac-Clc3s and VPS4(E228Q) induced vacuoles as Vac-VPS4(E228Q). (C) Average vacuole diameter per cell induced by Best3V2, Clc3s and VPS4(E228Q) 24 h after transfection in Hela cells. 50 cells with vacuoles were counted. (D**)** Vacuole size at 12 h, 24 h, 36 h, 48 h, 60 h and 84 h after transfecting of Best3V2 in Hela cells. (E) Percentage of Cells committing vacuole fusion (from 24 h to 48 h after transfection for Best3V2 and Clc3s, from 12 h to 36 h for VPS4(E228Q)) (F) Lifetime of Vac-V2 counted at 6 h interval which started from 12 h after transfection in Hela, compared to the lifetime of Vac-Clc3s and Vac-VPS4(E228Q) in Hela. Due to the short lifetime, the counting interval for Vac-VPS4(E228Q) was 3 h. (G) Apoptosis or necrosis induced by Vac-V2 (48 h after transfection), Clc3s (48 h after transfection) and VPS4(E228Q) (36 h after transfection). The apoptosis and necrosis assay was performed on live cells by FITC-Annexin V and PI double staining before being subjected to flow cytometry assay. 200 cells were counted for vacuole size and diameter. All error bars denote S.D. values. **, $p<0.01$, ***, $p<0.005$, ****, $p<0.001$, ****, $p<0.0005$. Scale bar, 20 μm.

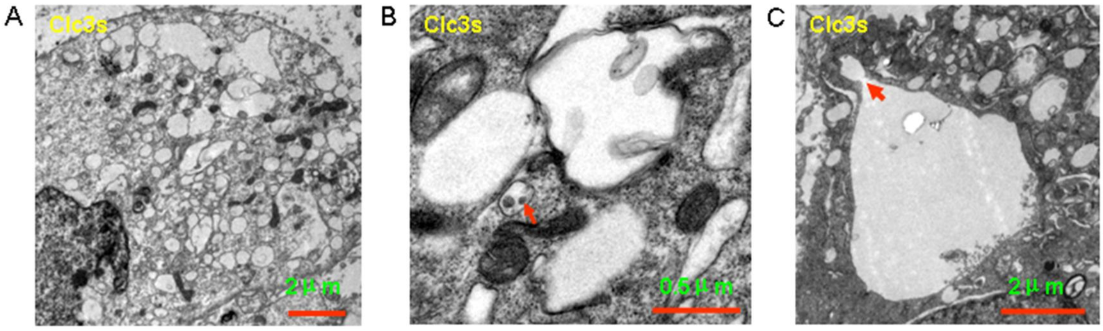

Figure S2: The morphology and the fusion between Clc3s vacuoles showed by TEM (A) Representative TEM showed Clc3s induced vacuoles (36 h) were filled with a number of small vacuoles and also with larger vacuoles in irregular shape. (B) Dense lysosome granules were only found in small Vac-Clc3s. (C) The vacuole fusion between Vac-Clc3s. Arrow showed the fusion of one small vacuole with a larger vacuole.
